# Mid-arm circumference, body fat, nutritional and inflammatory biomarkers, blood glucose, dialysis adequacy influence all-cause mortality in hemodialysis patients: A prospective cohort study

**DOI:** 10.1097/MD.0000000000014930

**Published:** 2019-03-22

**Authors:** Tuyen Van Duong, Pei-Yu Wu, Te-Chih Wong, Hsi-Hsien Chen, Tso-Hsiao Chen, Yung-Ho Hsu, Sheng-Jeng Peng, Ko-Lin Kuo, Hsiang-Chung Liu, En-Tzu Lin, Yi-Wei Feng, Shwu-Huey Yang

**Affiliations:** a School of Nutrition and Health Sciences, Taipei Medical University; b Department of Nutrition and Health Sciences, Chinese Culture University; c School of Medicine, Taipei Medical University; d Department of Nephrology, Taipei Medical University Hospital; e Department of Nephrology, Taipei Medical University-Wan Fang Hospital; f Division of Nephrology, Department of Internal Medicine, Taipei Medical University- Shuang Ho Hospital; g Division of Nephrology, Cathay General Hospital; h Division of Nephrology, Taipei Tzu-Chi Hospital, New Taipei 231; i Department of Nephrology, Wei Gong Memorial Hospital, Miaoli 351; j Department of Nephrology, Lotung Poh-Ai Hospital, Yilan 265; k Research Center of Geriatric Nutrition, Taipei Medical University; l Nutrition Research Center, Taipei Medical University Hospital, Taipei 110, Taiwan.

**Keywords:** all-cause mortality, body fat, dialysis adequacy, hyperglycemia, mid-arm circumference, nutritional and inflammatory bio-markers

## Abstract

Hemodialysis patients are at the high risk for morbidity and mortality. Evaluation and management of body composition and biochemical values are important to improve dialysis outcomes. We aimed to examine the effects of the mid-arm circumference, body fat, nutritional and inflammatory biomarkers, blood glucose, and dialysis adequacy on the mortality.

A prospective cohort study was conducted on 375 patients from 7 hospital-based dialysis centers. At baseline between September 2013 and April 2017, we assessed patients’ characteristics using chart review, body composition using the bioelectrical impedance analysis, and biochemical parameters using available laboratory tests. Patients were followed-up for all-cause mortality until April 2018. Kaplan–Meier Curves with Log-rank test, and Cox proportional hazards models were used to analyze the effects of assessed factors on the mortality.

During the median of follow-up time of 1.4 (1.0–3.2) years, 47 (12.5%) patients died. In the multivariate analysis, mid-arm circumference (hazard ratio, HR, 0.90; 95% confidence interval, 95%CI, 0.82–0.99; *P* = .036), body fat mass (HR, 0.95; 95%CI, 0.91–1.00; *P* = .031), percent body fat (HR, 0.96; 95%CI, 0.92–0.99; *P* = .024), serum creatinine (HR, 0.81; 95%CI, 0.68–0.96; *P* = .015), and eKt/V (HR, 0.07; 95%CI, 0.01–0.33; *P* = .001) reduced the mortality risk. Inflammation (HR, 2.90; 95%CI, 1.59–5.27; *P* < .001), hyperglycemia (HR, 2.16; 95%CI, 1.06–4.40; *P* = .033), and low serum uric acid (HR, 2.22; 95%CI, 1.15–4.31; *P* = .018) increased the death risk.

In hemodialysis patients, the higher values of the mid-arm circumference, body fat, serum creatinine, uric acid, and dialysis adequacy were associated with lower mortality, whereas, inflammation and hyperglycemia associated with higher mortality.

## 1 Introduction

The prevalence of patients with end-stage renal disease (ESRD) has been increasing in every country. These patients are at high risk for morbidity and mortality which further cause the social and economic burden to the country.^[1]^ In Taiwan, the prevalence of ESRD patients who underwent dialysis treatment was 3093 per million population in 2014. Ninety percent of those patients received in-center hemodialysis treatment.^[1]^ The older age, diabetes mellitus, cardiovascular diseases, inflammation, low nutritional status, anemia were summarized as the main causes of death in the ESRD patients undergoing hemodialysis treatment.^[2]^

Body fat indicators as the markers of obesity are strongly associated with several adverse outcomes such as insulin resistance, and diabetes in the general population,^[3]^ and in hemodialysis patients,^[4]^ in turn, increase the cardiovascular events in hemodialysis patients.^[5]^ Obesity and low muscle mass are the risk factors for mortality in the general population.^[6]^ The elevated waist circumference as a proxy for abdominal obesity is a risk for mortality in a number of studies.^[7,8]^ Conversely, other researchers found that body fat indicators showed the protective effects on the mortality, such as patients with low total body fat had a higher risk of death,^[9]^ low percent body fat increase the death risk,^[10]^ low fat tissue index (fat tissue/height^2^) strongly predicted mortality.^[11]^ On the other hand, higher muscle mass improves the survival of hemodialysis patients.^[11]^

In addition, several biochemical parameters were summarized as the strong predictors of mortality in hemodialysis patients.^[2,12,13]^ Several biomarkers of nutritional status such as albumin, creatinine,^[14]^ serum uric acid,^[13]^ cholesterol,^[12]^ the elevated C-reactive protein, high blood sugar,^[2,12,15]^ were shown as the strong predictors of mortality.

The evaluation and management of both body composition and biochemical parameters are highly important to reduce the mortality in hemodialysis patients. We aimed to investigate the impact of body composition and biochemical values on all-cause mortality in these patients. We hypothesized that hemodialysis patients with a better condition of body composition, and biochemical values would have better survival outcomes.

## 2 Methods

### 2.1 Study design

A prospective cohort study was conducted in 7 hospital-based dialysis centers in Taiwan. Patients were assessed at the baseline between September 2013 and April 2017 and followed-up until April 2018.

### 2.2 Study sample

**Figure 1:**
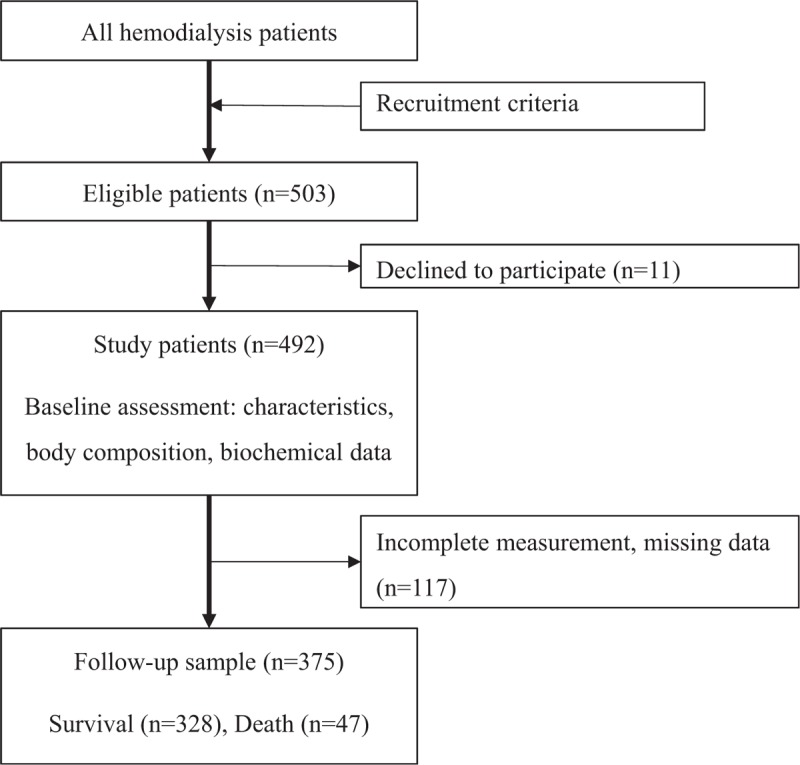
Flow chart of patients sampling and study procedure.

Hemodialysis patients were recruited from dialysis centers from Taipei Medical University Hospital (one group collected from September to December 2013; another group collected from November 2016 to January 2017), Taipei Medical University – Wan Fang Hospital (patients collected from April to May 2014), Taipei Medical University – Shuang Ho Hospital (patients collected in December 2014), Cathay General Hospital (patients collected in March 2016), and Taipei Tzu-Chi Hospital (patients collected in November 2016), Wei-Gong Memorial Hospital (patients collected from February to March 2017), and Lotung Poh-Ai Hospital (patients collected in April 2017). Patients’ recruitment criteria were described previously.^[16]^ A sample of 492 patients was assessed at the baseline, 375 patients with full assessments were followed-up for all-cause mortality which was depicted in Figure 1.

### 2.3 Measurements

At baseline, patients’ characteristics, body composition, and biochemical parameters were evaluated. Patients were then followed-up for all-cause mortality.

### 2.4 Patients’ characteristics

The information related to patients’ age, gender, hemodialysis vintage, Charlson comorbidity index,^[17]^ height, weight, body mass index, BMI (kg/m^2^) was collected using medical records. The physical activity level was assessed using the short version of the International Physical Activity Questionnaire.^[18]^ The physical activity score was the sum of minutes spent on vigorous, moderate, walking, and sitting activities over the last 7 days multiplied by 8.0, 4.0, and 3.3, 1.0, respectively.^[19]^ The metabolic equivalent task scored in minutes per week (named as MET-min/wk) was used to represent the physical activity.^[20]^

### 2.5 Body composition assessment

Body composition was assessed using the bioelectrical impedance analysis device using multiple operating frequencies of 1, 5, 50, 250, 500, and 1,000 kHz (InBody S10, Biospace, Seoul, Korea). The parameters were measured including soft lean mass (SLM), fat free mass (FFM), skeletal muscle mass (SMM), trunk lean mass (TrLM), right arm lean mass (RALM), left arm lean mass (LALM), right leg lean mass (RLLM), left leg lean mass (LLLM), mid-arm muscle circumference (MAMC), mid-arm circumference (MAC), waist circumference (WC), visceral fat area (VFA), body fat mass (BFM), percent body fat (PBF). Appendicular skeletal muscle mass (ASM) was the sum of RALM, LALM, RLLM, and LLLM.

### 2.6 Biochemical parameters

The blood samples were collected at the beginning of the first dialysis session of the week, then analyzed in the hospital laboratory. The biochemical parameters were assessed including high sensitive C-reactive protein (hs-CRP), Hemoglobin (Hgb), fasting plasma glucose (FPG), fasting plasma insulin (FPI), triglyceride (TG), high density lipoprotein cholesterol (HDL-C), low density lipoprotein cholesterol (LDL-C), total cholesterol (TC), serum calcium (Ca), serum phosphate (PO4), intact parathyroid hormone (iPTH), Homocysteine (Hcy), Albumin, pre-dialysis blood urea nitrogen (Pre-BUN), Creatinine, serum potassium (K), serum uric acid (SUA), dialysis adequacy (equilibrated Kt/V).

The biochemical parameters were categorized as inflammation if hs-CRP > 0.5 mg/dl),^[21]^ anemia if Hgb < 11 g/dl,^[22]^ elevated fasting plasma glucose if FPG ≥ 100 mg/dl, elevated insulin if FPI ≥ 12 μU/ml),^[23]^ dyslipidemia if TG ≥ 150 mg/dl, HDL-C < 40 mg/dl for men or HDL-C < 50 mg/dl for women, LDL-C ≥ 100 mg/dl, and TC ≥ 200 mg/dl).^[24,25]^ The serum Ca was classified into low (Ca < 8.4 mg/dl), normal (Ca 8.4–9.5 mg/dl), and high (Ca > 9.5 mg/dl). The serum phosphorus (PO_4_) is also classified into low level (PO4 < 3.5 mg/dl), normal (PO_4_ 3.5–5.5 mg/dl), and high (PO4 > 5.5 mg/dl). Calcium-phosphorus product is classified into normal (Ca × PO_4_ < 55 mg^2^ /dl^2^), and high (Ca × PO_4_ ≥ 55 mg^2^ / dl^2^). The iPTH was classified into normal (iPTH 150–300 pg/ml), and high (iPTH ≥ 300 pg/ml).^[26]^ Homocysteine > 14 μmol/L was defined as hyperhomocysteinemia,^[27]^ serum potassium ≥ 5.0 mEq/L was defined as hyperkalemia.^[28]^ Serum uric acid was classified into lowest quintile, middle 3 quintiles, and highest quintiles.^[29]^

### 2.7 Statistical analysis

Descriptive analysis was utilized to describe the distribution of studied variables. The variables with approximately normal distribution were reported as mean ± standard deviation (SD), otherwise, median (interquartile range) was presented. Categorical variables were presented as frequency and percentage. The ANOVA, Mann–Whitney *U* test, and Chi-Squared test were used appropriately to compare characteristics, body composition, and biochemical parameters between survival group and non-survival one.

Cox proportional hazard models were used to examine the effects of body composition, and biochemical parameters on mortality. Model 1 examined the bivariate effects of body composition, biochemical parameters on mortality. Model 2 were adjusted for patients’ age, gender, hemodialysis vintage, Charlson comorbidity index, and physical activity. Model 3 included variables in model 2, plus biochemical parameters, and body composition which showed significant effects on all-cause mortality in model 1. Hazard ratios and 95% confidence intervals were reported. The Kaplan–Meier curves, and Log-rank test were used to elucidate the survival probability by variables of body composition and biochemical parameters.

All data analysis was conducted using the IBM SPSS software version 20.0 for Windows (IBM Corp., New York, USA). The statistically significant level was set at *P* value < .05.

### 2.8 Ethical approval

The study was conducted in accordance with the Declaration of Helsinki, and the protocol was approved by the Ethics Committee of Taipei Medical University Joint Institutional Review Board (TMU-JIRB No. 201302024) for conducting the study in Taipei Medical University Hospital, Wan-Fang Hospital, Shuang Ho Hospital, Wei-Gong Memorial Hospital; the ethical committee of Cathay General Hospital (CGH-OP104001) for conducting the study in Cathay General Hospital, and Lotung Poh-Ai Hospital; and in Taipei Tzu-Chi Hospital (04-M11-090). Patients had signed the informed consent forms before study conducted.

## 3 Results

Patients aged 60.6 ± 11.9 years, 34.9% aged 65 years and above, 57.1% were men. The mean of hemodialysis vintage, Charlson comorbidity index, physical activity score, and BMI were 5.6 ± 4.9 years, 4.7 ± 1.6 score, 4905.0 ± 1884.4 MET-min/wk, 23.5 ± 3.9 kg/m^2^, respectively. There were 40.5% of patients with overweight and obese. The median of follow-up time was 1.4 (1.0–3.2) years, 47 (12.5%) patients died (Table 1).

**Table 1:**
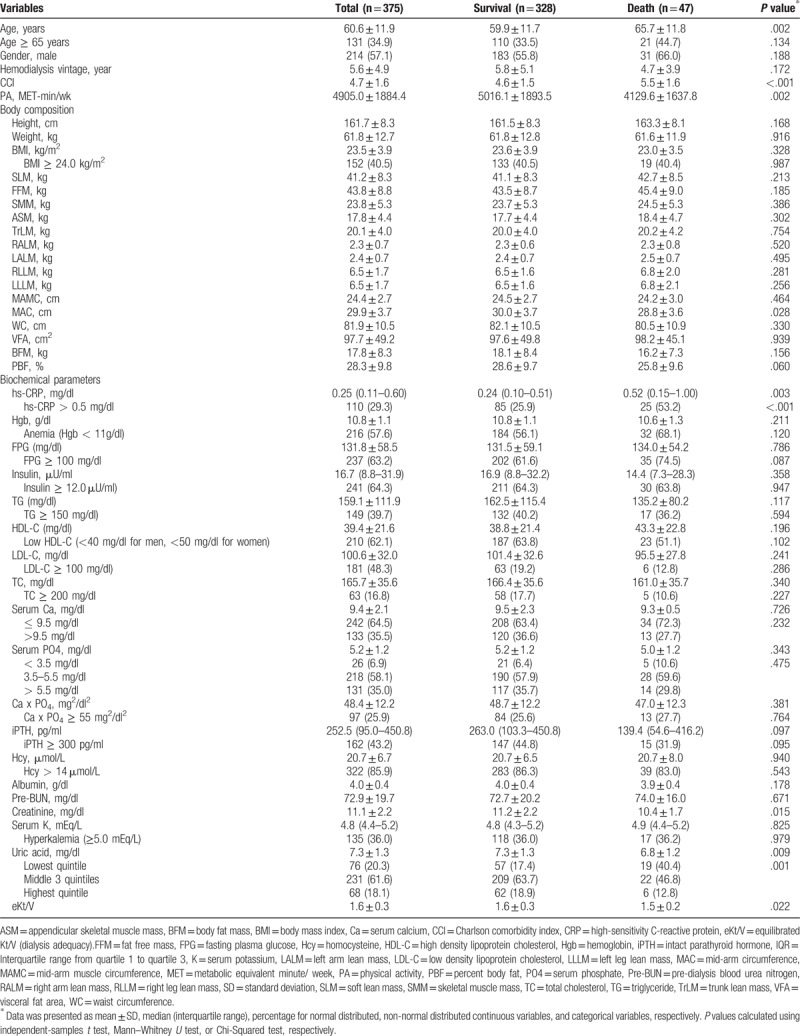
Patients’ characteristics, body composition, biochemical parameters at the baseline.

Patients who died during the follow-up were with older age (*P* = .002), higher comorbidity index (*P* < .001), lower physical activity level (*P* = .002), lower MAC (*P* = .028), higher inflammation (*P* < .001), lower serum creatinine (*P* = .015), lower eKt/V (*P* = .022), and lower serum uric acid (SUA) (*P* = .009; Table 1).

The results of bivariate analysis showed that the factors significantly influenced the mortality were MAC (hazard ratio, HR, 0.92; 95% confidence interval, 95%CI, 0.85–0.99; *P* = .034; Table 2), elevated hs-CRP (HR, 3.01; 95%CI, 1.70–5.34; *P* < .001), high FPG (HR, 1.98; 95%CI, 1.03–3.83; *P* = .042), serum creatinine (HR, 0.81; 95%CI, 0.70–0.94; *P* = .005), low SUA (HR, 2.67; 95%CI, 1.49–4.78; *P* = .001), and eKt/V (HR, 0.95; 95%CI, 0.91–1.00; *P* = .031; Table 3).

**Table 2:**
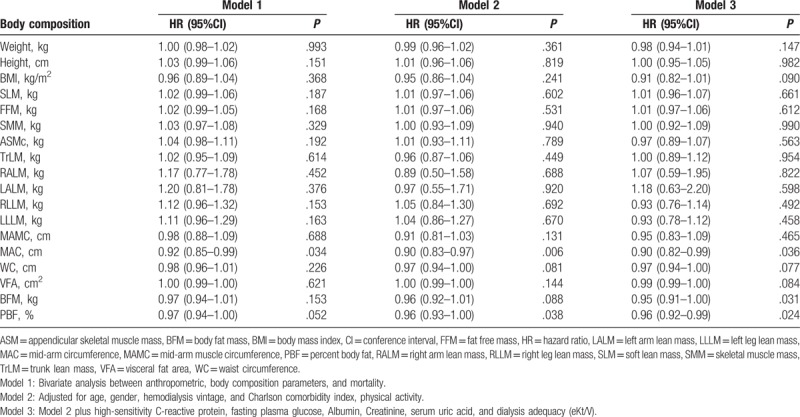
Body composition and hazard ratio of mortality among hemodialysis patients (n = 375).

**Table 3:**
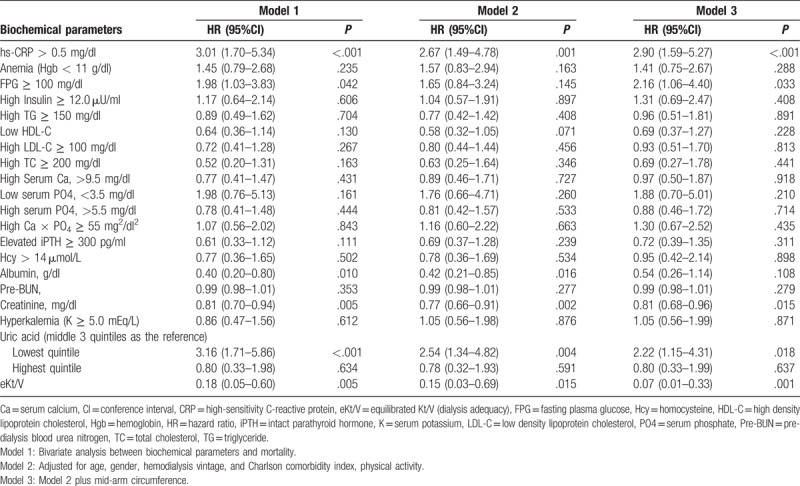
Biochemical indicators and hazard ratio of mortality among hemodialysis patients (n = 375).

Survival plots (Kaplan–Meier Curves) are shown in Figure 2  . The survival was not significantly different between the tertiles of MAC (*P* = .186; Fig. 2  A). The survival rate was significantly lower in elevated hs-CRP (*P* < .001; Fig. 2  B), elevated FPG (*P* = .037; Fig. 2  C). The survival rate was significantly different between tertiles of serum creatinine (*P* = .040; Fig. 2D), tertiles of the SUA (*P* < .001; Fig. 2E), and tertiles of eKt/V (*P* = .043; Fig. 2F).

**Figure 2:**
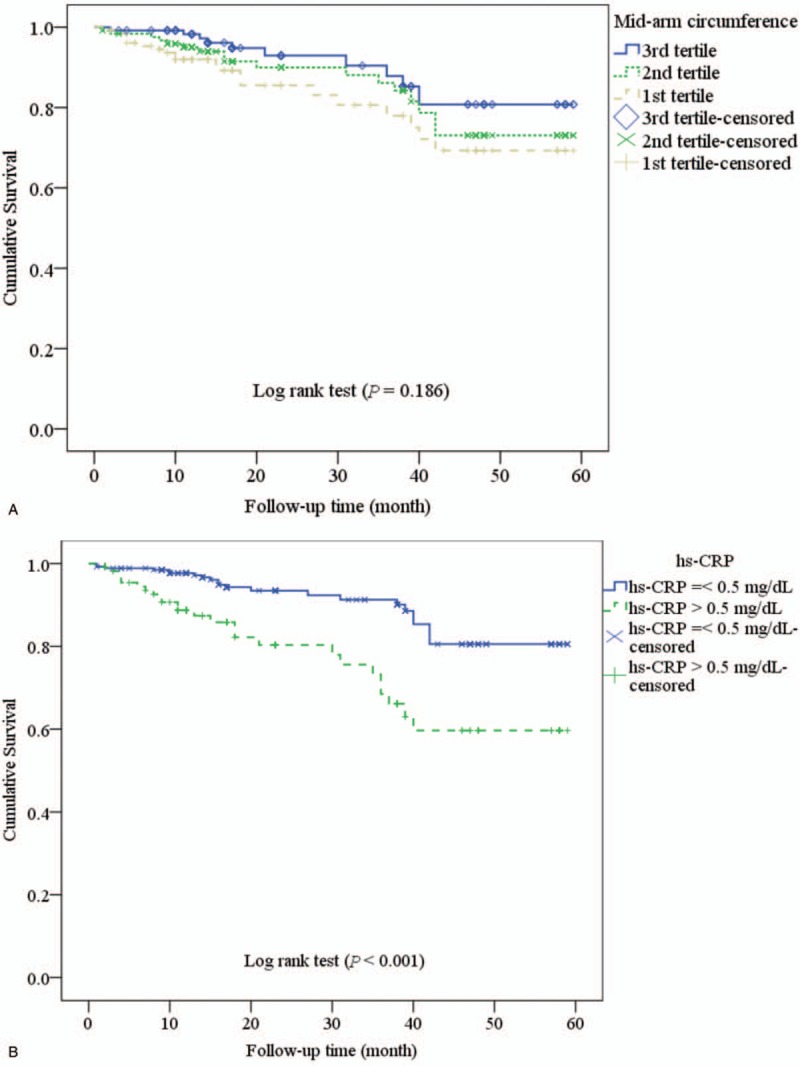
Survival plots (Kaplan–Meier Curves) according to the tertile of mid-arm circumference (A), elevated high-sensitivity C-reactive protein (hs-CRP) (B), high fasting plasma glucose (C), tertile of serum creatinine (D), tertile of serum uric acid (E), and equilibrated Kt/V (F).

**Figure 2 (Continued):**
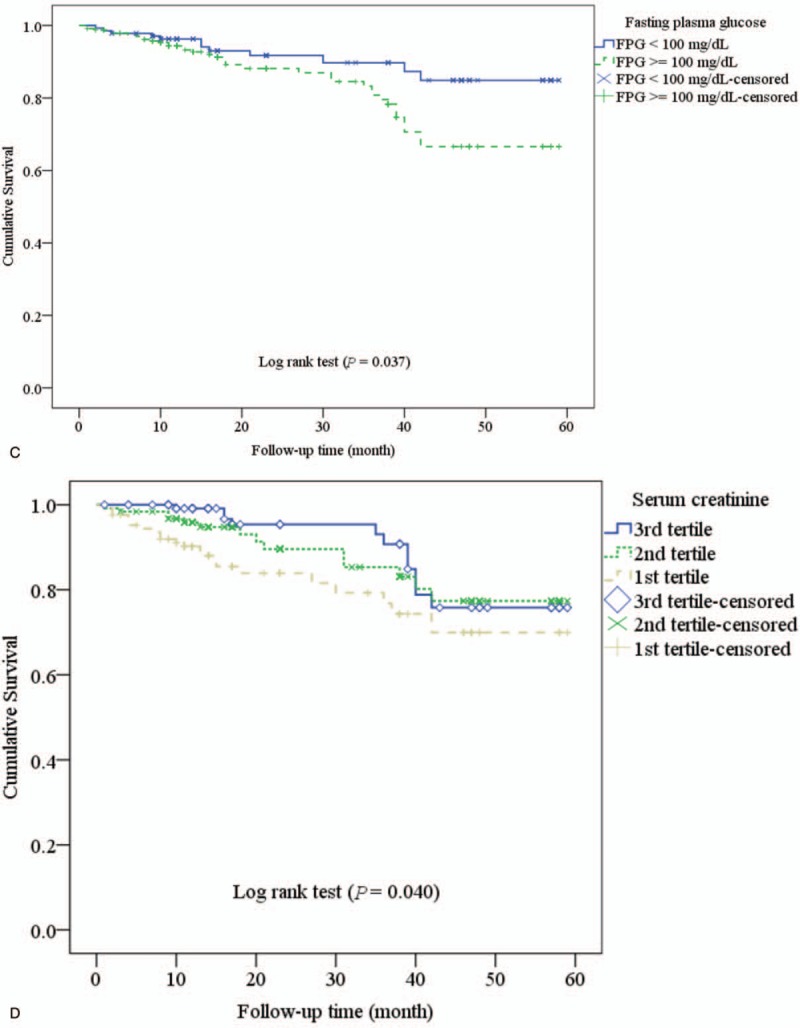
Survival plots (Kaplan–Meier Curves) according to the tertile of mid-arm circumference (A), elevated high-sensitivity C-reactive protein (hs-CRP) (B), high fasting plasma glucose (C), tertile of serum creatinine (D), tertile of serum uric acid (E), and equilibrated Kt/V (F).

**Figure 2 (Continued):**
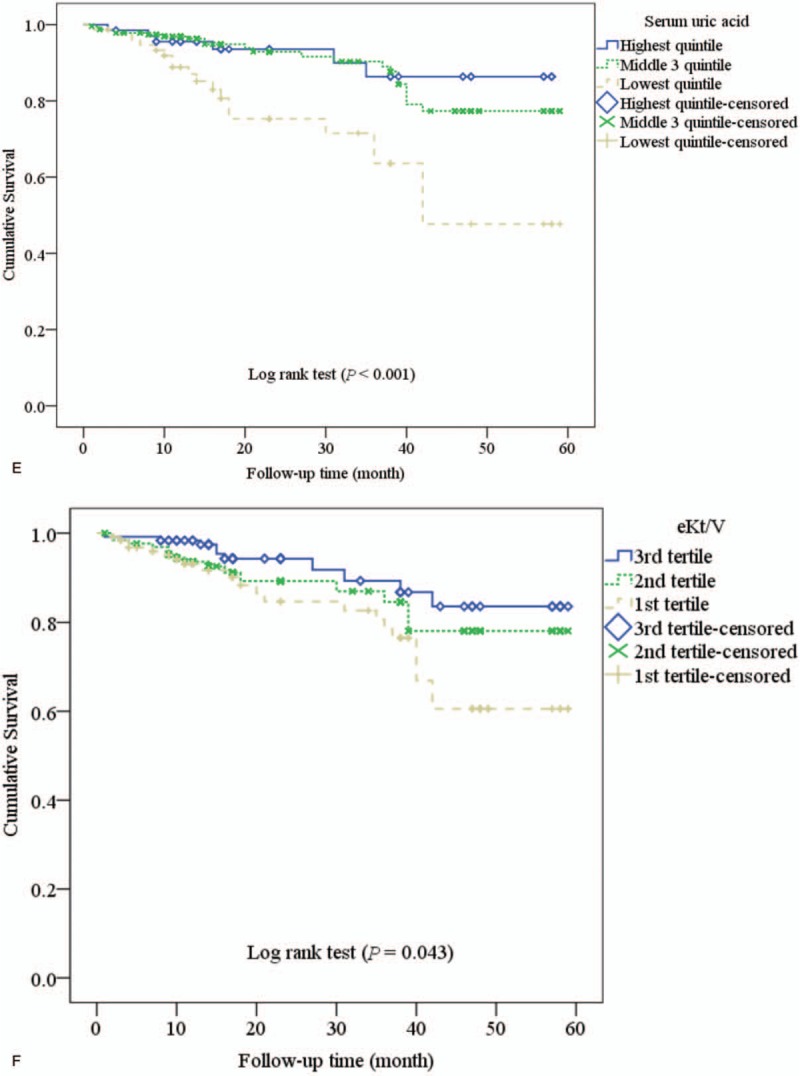
Survival plots (Kaplan–Meier Curves) according to the tertile of mid-arm circumference (A), elevated high-sensitivity C-reactive protein (hs-CRP) (B), high fasting plasma glucose (C), tertile of serum creatinine (D), tertile of serum uric acid (E), and equilibrated Kt/V (F).

In the multivariate analysis (model 2), the predictors of all-cause mortality regarding body composition were MAC (HR, 0.90; 95%CI, 0.83–0.97; *P* = .006), PBF (HR, 0.96; 95%CI, 0.93–1.00; *P* = .038). After adjusted for patients’ characteristics and significant biochemical parameters (hs-CRP, FPG, albumin, creatinine, SUA, and eKt/V) in model 3, MAC (HR, 0.90; 95%CI, 0.82–0.99; *P* = .036), PBF (HR, 0.96; 95%CI, 0.92–0.99; *P* = .024) remained the protective effect on all-cause mortality. In addition, BFM (HR, 0.95; 95%CI, 0.91–1.00; *P* = .031) significantly reduced all-cause mortality. The other body composition indicators did not show the significant effects on all-cause mortality among hemodialysis patients (*P* > .05; Table 2).

Regarding the effect of biochemical values on mortality, the results in model 2 showed that elevated hs-CRP (HR, 2.67; 95%CI, 1.49–4.78; *P* = .001), and low SUA (HR, 2.54; 95%CI, 1.34–4.82; *P* = .004) significantly increased all-cause mortality risk. Serum albumin (HR, 0.42; 95%CI, 0.21–0.85; *P* = .016), serum creatinine (HR, 0.77; 95%CI, 0.66–0.91; *P* = .002), and eKt/V (HR, 0.15; 95%CI, 0.03–0.69; *P* = .015) significantly reduced all-cause mortality risk. In model 3, elevated hs-CRP (HR, 2.90; 95%CI, 1.59–5.27; *P* < .001), high FPG (HR, 2.16; 95%CI, 1.06–4.40; *P* = .033), and low SUA (HR, 2.22; 95%CI, 1.15–4.31; *P* = .018) significantly increased the risk of death, while serum creatinine (HR, 3.16; 95%CI, 1.71–5.86; *P* < .001), and eKt/V (HR, 0.18; 95%CI, 0.05–0.60; *P* = .005) showed the significant protective effect on mortality among hemodialysis patients. Other biochemical parameters did not show the significant effects on mortality among hemodialysis patients (*P* > .05; Table 3).

## 4 Discussion

The current study showed that mid-arm circumference, body fat mass, percent body fat, serum creatinine, uric acid, hs-CRP, fasting plasma glucose, and eKt/V significantly associated with all-cause mortality in hemodialysis patients.

The current findings confirmed that the mid-arm circumference (MAC) but not the mid-arm muscle circumference (MAMC) demonstrated a significant effect on the mortality. However, the MAMC was shown as an accurate predictor of mortality in the previous studies.^[30,31]^ In addition, our study illustrated that body fat mass, percent body fat strongly predicted the mortality in hemodialysis patients. This additionally contributed to literature regarding the protective effect of body fat on the mortality among the end-stage renal disease patients receiving hemodialysis treatment.^[9,11,32]^

In the present study, all the lean mass indicators were not associated with all-cause mortality. The finding was inconsistent with a previous study with more than 4 years of follow-up which showed that lower thigh muscle mass associated with higher mortality in hemodialysis patients.^[33]^ Another study elucidated the association between lean body mass and survival among patients undergoing hemodialysis in different race and ethnicity.^[34]^ Low lean body mass also showed a strong association with a higher rate of hospitalization and mortality in hemodialysis patients in a long-term follow-up study.^[30]^ This inconsistency might be due to the short follow-up time in the current study.

In the current study, waist circumference, and the visceral fat area were not significantly associated with all-cause mortality. However, in a previous study, abdominal fat consisting of the visceral fat area and subcutaneous fat area showed the protective effects on the mortality in patients undergoing hemodialysis.^[35]^

The current study showed that the high hs-CRP significantly increased 2.9 folds of death risk, and the elevated fasting plasma glucose increased more than 2 times risk of mortality. In Western and Eastern countries, hemodialysis patients with high hs-CRP, and diabetes had 1.25 to 1.68 times, and 2.00 to 2.08 times risk of death, respectively.^[2]^ The effect of elevated CRP on mortality was also found in the international Monitoring Dialysis Outcome Initiative cohort in 16 European countries,^[36]^ in the Dialysis Outcomes and Practice Patterns Study in Japan,^[15]^ and in South Korea.^[37]^

Serum albumin significantly reduced all-cause mortality risk by about 58% in model 2, but the effect was attenuated to 46% and not significant in model 3. A systematic review showed that patients with high serum albumin level had about 48% lower mortality risk as compared to those who with low albumin level, in both Western and Eastern countries.^[2]^ After adjusted for arm circumference in model 3, the effect of serum albumin on all-cause mortality was disappeared, but the effect of elevated fasting plasma glucose on the mortality turned to be significant. This might show the potential interactions between mid-arm circumference and fasting plasma glucose or serum albumin. Therefore, the evaluation of those parameters is critically important in predicting all-cause mortality among hemodialysis patients.

Serum creatinine as a nutritional biomarker was presented as a protective factor of mortality in the current study which significantly reduced 19% mortality risk. The previous *Q*-Cohort study in Japan also showed that lower creatinine index associated with high all-cause mortality risk in hemodialysis patients.^[38]^ On the other hand, patients with low level of serum uric acid had more than 2 times hazard of mortality as compared to those who with the normal level in the present study. This finding was consistent with a previous study which reported that low serum uric acid independently predicted the all-cause mortality in hemodialysis patients.^[39]^ In addition, the serum uric acid has been known as a nutritional biomarker and predictor of dialysis outcomes,^[40]^ predicted cardiovascular mortality,^[41]^ and all-cause mortality among hemodialysis patients.^[40,41]^ In the present study, as compared to patients with normal serum uric acid level, those who with low serum uric acid level, but not with high serum uric acid level had high mortality risk. This was supported by a previous study in the United States.^[42]^ However, in the literature, both low and high level of uric acid predicted higher mortality risk.^[29]^

In the present study, dialysis adequacy or eKt/V significantly reduced about 93% mortality risk after adjusted for confounders. This was in the line with the Dialysis Outcomes and Practice Patterns Study (DOPPS) which showed that low Kt/V was associated with higher mortality in hemodialysis patients.^[43]^

The current study demonstrated a limitation related to small sample size which the interactions between body composition indicators and biochemical parameters were not examined. The future study with the larger sample size is required. Another limitation was that we did not access the medications used for study patients, which might confound the associations. This limitation could be avoidable as patients have received similar treatment and follow-up under the regulation of the National Health Insurance program in Taiwan.^[44]^ Finally, the follow-up time was varied between group of patients in multiple dialysis centers from 1 year to 5 years, which was disequilibrium for observation, and should exist bias. Future studies are required for confirming the current findings. The strength of this study lay on the objective measurements of body composition indicators and biochemical parameters.

## 5 Conclusion

This was a comprehensive study to examine the effects of body composition and biochemical parameters on all-cause mortality in hemodialysis patients. The results showed the protective effects of the mid-arm circumference, body fat mass, percent body fat, serum creatinine, uric acid, and dialysis adequacy (eKt/V), whereas, the inflammation and hyperglycemia presented as the risks for all-cause mortality. The evaluation of body composition and laboratory parameters could contribute to improving the dialysis outcomes among patients receiving the hemodialysis treatment.

## Acknowledgments

The authors thank all medical staff and patients who participated in the study from Taipei Medical University Hospital, Wan-Fang Hospital, Shuang Ho Hospital, Cathay General Hospital, and Taipei Tzu-Chi Hospital, Wei-Gong Memorial Hospital, and Lutong Poh-Ai Hospital. The authors also thank Chi-Sin Wang, I-Hsin Tseng, Tai-Yue Chang for helping with data collection.

## Author contributions

TVD contributed to conception and design, consulted a statistician, analyzed the data, interpreted the results and drafted the manuscript. PYW, TCW, HHC, THC, YHH, SJP, KLK, HCL, and ETL contributed to study design, acquisition of data, and investigation. YWF, contributed to the methodology, data collection, interpretation of data, and discussion. SHY contributed to the conception, overall study design and critically revised the manuscript. All authors read and approved the final version of the manuscript.

**Conceptualization:** Tuyen Van Duong, Pei-Yu Wu, Te-Chih Wong, Hsi-Hsien Chen, Tso-Hsiao Chen, Yung-Ho Hsu, Sheng-Jeng Peng, Ko-Lin Kuo, Hsiang-Chung Liu, En-Tzu Lin, Shwu-Huey Yang.

**Data curation:** Pei-Yu Wu, Te-Chih Wong, Hsi-Hsien Chen, Tso-Hsiao Chen, Yung-Ho Hsu, Sheng-Jeng Peng, Ko-Lin Kuo, Hsiang-Chung Liu, En-Tzu Lin, Yi-Wei Feng, Shwu-Huey Yang.

**Formal analysis:** Tuyen Van Duong.

**Funding acquisition:** Shwu-Huey Yang.

**Investigation:** Pei-Yu Wu, Te-Chih Wong, Hsi-Hsien Chen, Tso-Hsiao Chen, Yung-Ho Hsu, Sheng-Jeng Peng, Ko-Lin Kuo, Hsiang-Chung Liu, En-Tzu Lin, Yi-Wei Feng, Shwu-Huey Yang.

**Methodology:** Tuyen Van Duong, Pei-Yu Wu, Te-Chih Wong, Hsi-Hsien Chen, Tso-Hsiao Chen, Yung-Ho Hsu, Sheng-Jeng Peng, Ko-Lin Kuo, Hsiang-Chung Liu, En-Tzu Lin, Yi-Wei Feng, Shwu-Huey Yang.

**Project administration:** Yi-Wei Feng.

**Resources:** Shwu-Huey Yang.

**Software:** Tuyen Van Duong.

**Supervision:** Shwu-Huey Yang.

**Validation:** Tuyen Van Duong.

**Visualization:** Tuyen Van Duong.

**Writing – original draft:** Tuyen Van Duong.

**Writing – review & editing:** Tuyen Van Duong, Shwu-Huey Yang.

Shwu-Huey Yang orcid: 0000-0002-3707-1166.
